# Creating Community and Engaging Community: The Foundations of the Estate Little Princess Archaeology Project in St. Croix, United States Virgin Islands

**DOI:** 10.1007/s10761-021-00600-z

**Published:** 2021-05-19

**Authors:** Ayana Omilade Flewellen, Alicia Odewale, Justin Dunnavant, Alexandra Jones, William White

**Affiliations:** 1 Department of Anthropology, University of California, Riverside, USA; 2 Department of Anthropology, University of Tulsa, Tulsa, OK USA; 3 Department of Anthropology, University of California, Berkeley, CA USA; 4 Department of Anthropology, Vanderbilt University, Nashville, TN USA; 5 Archaeology in the Community, Washington, DC USA

**Keywords:** African diaspora archaeology, Community based participatory research, St. Croix, US Virgin Islands, Archaeological field school

## Abstract

This article discusses how Co-Principal Investigators that designed and executed the Estate Little Princess Archaeology Project (ELPAP) came together as a community, to demonstrate how such a formation within the discipline, with all its ups and downs, facilitates the skills needed to conduct community archaeology. By using the ELPAP as a case study, this article provides a multiscale examination of the ELPAP, expanding the discourse on community archaeology to include community building practices among archaeologists, between organizations, and with communities impacted by archaeological work.

## Introduction

Most literature on community-engaged archaeology, rightly so, focuses on the relationship between the archaeologists and the communities impacted by archaeological work. However, work through the Estate Little Princess Archaeology Project (ELPAP) demonstrates that collaboration among the co-Principal Investigators (PIs) is equally important for successful community collaboration to occur. Community archaeology is the practice of using archaeological methods to address issues of importance in collaboration with the community, broadly defined. Community archaeology seeks to incorporate local, descended, and stakeholder communities in all aspects of the archaeological enterprise (Marshall 2002; Moser et al. 2002).

In order to truly understand what community archaeology entails, it is important to define community. In this article we stray away from terms like “public” and “local,” which are often very broad, and instead lean into community as a representation of an intimate group of people. We define a community as a social group of varied size who share common characteristics and are perceived to belong to a distinct segment of society. Though a community is one unit, an archaeological site generally has more than one community that can lay historical claims to the site. Doing community archaeology means grappling with these dynamics. By discussing how we, as co-PIs, came together as a community we hope to demonstrate how such a formation, with all its ups and downs, facilitates the skills needed to conduct community archaeology.

Archaeological work at the Estate Little Princess (ELP) on the island of St. Croix, US Virgin Islands (USVI), began as an idea in 2016 when Jay Haigler, a Board Member of Diving With a Purpose, contacted one of the authors and co-founder of the Society of Black Archaeologists (SBA), about a possible collaboration with the Slave Wrecks Project (SWP) on a joint community collaborative maritime and terrestrial archaeology project on the island of St. Croix, USVI. Diving With a Purpose is a non-profit organization dedicated to oceanic conservation and the preservation of maritime heritage sites, specifically those pertaining to the African Diaspora. The idea that was sowed in 2016 blossomed into a multi-year project where five archaeologists - all people of African descent - came together as a community under the umbrella of the Society of Black Archaeologists to develop and implement a community-focused and collaborative archaeological project. The Estate Little Princess Archaeological Project (ELPAP), based at the ELP, is a project with Black archaeologists at the helm that is dedicated to training Black graduate and undergraduate students as well as students attending middle and high school in archaeological theory and method. A program like this, run by Black archaeologists designed to train Black students, is extremely rare, yet programs of this nature address the systemic lack of racial diversity in the field of archaeology by establishing new pathways for historically underrepresented students to break into the field.

Since 2016, the ELPAP has partnered with over a dozen organizations and cultural institutions and trained over 40 local youth between the ages of 11-16 and nine undergraduates from Historically Black Colleges and Universities (HBCUs) across the country, two of whom have been accepted into renowned graduate school programs for Anthropology and History. In addition to cultivating local youth and undergraduate archaeology scholars, the program is a successful training site for graduate students, where they gain supervisory field experience and essential support as they prepare to lead their own archaeology projects. At least four graduate students, outside of the previously mentioned nine HBCU students, have been able to leverage their connections to ELPAP to strengthen their own teaching and research agendas.

This article offers an introspective examination of how a community of archaeologists, all at different stages of their academic careers, came together carrying with them a myriad of archaeological methods, pedagogies, and epistemologies to do community archaeology as a means to address and push against harmful exclusionary practices in the field that marginalize Black and Brown scholars and communities.

The ELPAP expanded on archaeology projects in the 1990s where teams of mid-career and senior Black scholars in the field undertook career-shaping projects that ushered in training opportunities for junior Black scholars to gain a foothold in the field. Two projects that have developed in this manner include archaeological work at the Rich Neck Plantation and at the New York African Burial Ground Project (NYABG). While Marley R. Brown III was the PI for the Rich Neck Plantation project from June 1994 to February 1995, Maria Franklin and Anna Agbe-Davies, two Black women early in their archaeological careers, collectively led efforts to excavate the enslaved peoples’ quarters at the plantation site (Franklin 2004). It was during these field seasons that Whitney Battle-Baptiste and Ywone Edwards-Ingram also participated in research at the site making the Rich Neck Plantation the first archaeological site where four Black women - in various stages of their professional careers - worked alongside each other.

The project did not have a structured community-engagement aspect to it, but Franklin and Agbe-Davies undertook a tremendous amount of work forging connections between African American organizations and the project while also organizing several public education programs related to the excavation (Franklin pers. comm. 2020). Battle-Baptiste continued after this project to join Franklin as her first Ph.D. student at the University of Texas, and all four women have continued in the field as a community that provides support to each other as they navigate the discipline. All four of these women have since become established scholars in the field, revered for their ongoing contributions that expand the breadth of archaeological research on the African diaspora. What Franklin’s 1998 paper “Why Are There so Few Black American archaeologists?” discusses, and that numerous oral histories collected from archaeologists of African descent gathered by the SBA confirm nearly a decade later (Battle-Baptiste 2012a, 2012b; Brunache 2014; Jones 2014), is that representation in the field and mentorship provide pathways for Black people to access and navigate the field of archaeology. What work at the Rich Neck Plantation in the mid-1990s did, that work at the ELP now builds upon, is a tradition of Black archaeologists building community among themselves as a praxis that fosters community-engaged work.

The NYABG, an eighteenth-century African burial ground rediscovered underneath the heart of New York’s financial center in 1991 during a federal construction project, was another project that ushered in a watershed moment in the field of African American archaeology. Analysis of the human remains and reinterment were ultimately handled by Howard University and a diverse team of archaeologists of African descent. From the project a number of archaeologists of African descent, including Michael Blakey, Joe Joseph, Cheryl LaRoche, Warren Perry, and the late Mark E. Mack, crafted research agendas that not only propelled their careers in the field but answered critical questions around ancestry, enslavement, and the lived experiences of Africans in the Americas (e.g., LaRoche and Blakey 1997; Mack and Blakey 2004). It was also one of the few archaeological projects that thrived as a direct result of community activism (LaRoche 2011). Additionally, centering the analysis at Howard University’s Cobb Laboratory, the only Historically Black College and University (HBCU) with an anatomical collection, impacted a generation of students who witnessed the outpouring of community support and research that continues to result from the project. The project demonstrates that intentional collaborations amongst academics is imperative to a successful program that centers community engagement. Furthermore, both the field school at Rich Neck Plantation and the NYABG were foundational because at the time, there were fewer than five PhD-holding archaeologists of African descent in the field (Barbour 1994:12).

## Building the Capacity to Reclaim Heritage

The NYABG was initiated because of its nexus with historic preservation laws in the United States that mandate potentially historic properties like archaeological sites be identified before they are damaged by construction. The protests associated with the NYABG were motivated by the fact that archaeologists on the project, the majority of whom were white, failed to address the questions African Americans in New York City wanted to know about their ancestors (LaRoche and Blakey 1997). These protests were compounded by the fact that the archaeologists doing the fieldwork were almost all white (Frohne 2015). The vast majority of archaeology in the United States is conducted, identified, and evaluated under auspices of cultural resource management (CRM), which refers to the collective of historic preservation professionals, including archaeologists, who use their professional experience to make recommendations on historic properties based on their ability to convey significance as codified under historic preservation legislation (King 2013). Additionally, the laws underlying CRM archaeology, like the National Historic Preservation Act (NHPA) and National Environmental Policy Act (NEPA), are applicable to African diasporic sites with a Federal nexus throughout the United States and its territories but the way these laws are applied by archaeologists does not mandate consultation with local African American communities.

Despite the success won at the NYABG, CRM archaeology consistently fails to adequately interpret an unknown number of African diasporic sites with local significance due to the lack of consultation with local African American communities. Research conducted elsewhere in the United States has shown the process CRM archaeologists use to evaluate African American sites frequently overlook the significance of African diasporic sites to local Black communities (Babiarz 2011; Barile 2004). CRM archaeologists also recommend the preservation of European American sites more often than African American ones (Barile 2004). Even if African American communities are consulted about their heritage sites, there are few protocols in historic preservation laws to address the intangible cultural aspects of African diasporic sites, even though it is usually the intangible connection to these sites that provide African American communities with the strongest connection. For example, in St. Croix there is greater emphasis among Crucian heritage preservationists on protecting sites associated with a legacy of resistance and expressions of sacred culture that are traditionally marked by the presence of Baobab, Kapok or Jumbie, Crucian Mahogany, or Tamarind trees that all comprise a special category of spirit trees based on their African origins and association with the spirit world (Highfield 2018). While in contrast legal protections from government sources are extended mainly to sites with standing sugar mills and other spaces of colonial occupation in St. Croix, the same equitable protection is not extended to sites associated with extensive archival holdings, standing spirit trees or unmarked graves, or spaces with oral histories associated with independent Black markets, Free Black homesteads, Maroon villages, gathering spaces for dancing and retelling Caruso songs, sites burned down in the course of historic anti-slavery or anti-labor rebellions, or coastal spaces that marked the first steps of enslaved Afro-Caribbean ancestors into a new world, which would all arguably hold greater importance within the Crucian community than the protection of a sugar mill (Bastian 2003; Dunnavant et al. 2018; Flewellan 2019; Highfield 2018; Norton and Espenshade 2007; Odewale 2016). It is possible for local communities to use these same laws to preserve heritage sites on their own, but the process is complicated and depends on qualified professionals who have a combination of experience and education to be accepted by state historic preservation offices (King 2013).

The way historic preservation laws fail to serve African America can be partially remedied by providing ways for local communities to participate in the interpretation of their heritage sites. The Estate Little Princess is a historic property listed in the National Register of Historic Places (National Register) primarily for its architectural value as an example of a Danish sugar plantation (Wright et al. 1980). The village where enslaved Afro-Crucians lived is noted as a part of this site, but its cultural value to the local community has not been recognized. Archaeological remains were noted in the National Register form for the Estate, but the nature and extent of these resources was not known at that time. The nomination does not describe any of the cultural elements of the Estate that could have been conveyed by interviewing local Afro-Crucian residents. The current ELPAP has provided space for Afro-Crucian people to help interpret this site in their own terms, something that has been overlooked by previous historic preservationists.

The collaborative archaeology of the ELPAP seeks to help reclaim Afro-Crucian sovereignty over their own heritage sites, a goal of many non-white groups around the world. As described in more detail later in this text, aspects of this work parallel tenets of Indigenous archaeology, specifically archaeological research conducted by and for Indigenous people (Watkins 2001) and decolonized archaeological practice (Schneider and Hayes 2020). In the past, indigenous traditional knowledge was routinely co-opted without archaeologists fully understanding this knowledge or acknowledging its sources. Sacred objects, human remains, and other powerful artifacts were extracted from sites and curated in museums without Indigenous permission.

In the United States, the lack of protections, inadequate oversight, and avoidable destruction commonly happening to African American heritage sites today also happened to Native American sites on a more widespread scale until the passage of several key laws and executive orders with explicit provisions for protecting Native American sites. These laws include the American Indian Religious Freedom Act (1978), the Archaeological Resources Protection Act (1979), the Native American Graves Protection and Repatriation Act (NAGPRA) of 1990, and the Religious Freedom Restoration Act (1993). In 1992, the NHPA was amended to allow Native American tribes to create tribal historic preservation offices that could assume the functions of the state historic preservation office on their tribal lands (King 2013; Watkins 2001). Tribal cultural revitalization efforts, of which archaeological data is but one part, can be seen as a framework for organic heritage conservation advocacy for other disenfranchised, colonized peoples.

Current historic preservation laws allow federally recognized Native American tribes, Native Hawaiians, and Alaskan Natives to access sacred sites and mandate that government agencies consult with these groups on development projects with a federal nexus. However, these laws do not provide the same provisions for all Native American tribes or other non-white groups in the United States. Also, these laws simply require “consultation.” They do not mandate “collaboration” with Native American tribes, which means Indigenous sites had protections but did not provide sovereignty over cultural knowledge or access to sacred sites. Nevertheless, several tribal historic preservation offices (THPOs) have expanded their capacity to conduct cultural resources work. Archaeology has been integrated into tribal cultural revitalization programs and Native American tribes have increasingly attained the skills and knowledge to manage archaeological sites on their lands. Helping build the capacity for African Americans to better use historic preservation laws to advocate for their own heritage sites is another way archaeologists can help keep African diaspora sites from being overlooked, misinterpreted, or destroyed.

Cultural resource management archaeologists have been forced to interact with Native people in the United States because so much of archaeology is done through federal laws that mandate tribal consultation. While this has not automatically led to collaboration and equity between archaeologists and Native Americans, it has helped generate a new generation of archaeologists willing to listen to Native American tribes and other Indigenous people to provide space for their input in archaeological interpretations (Atalay 2006; Colwell-Chanthaphonh et al. 2010; Stapp and Burney 2002; Watkins 2001). Native American archaeologists have also pushed to use archaeology in ways that help teach students about Native American pasts (Supernaut 2020) while questioning whether archaeology needs to be “undisciplined” from its current manifestation if it is to be of use to Native American tribes (Schneider and Hayes 2020). In this way, Native American tribes have increased sovereignty over narratives associated with their pasts and are better able to access materials from their heritage sites while pushing for increased reflexivity among archaeologists. Growth of THPOs with the ability to conduct archaeology on their own terms is leading to situations where tribes are hiring white CRM archaeologists as their consultants and are reviewing their work to make sure it dovetails with tribal preservation goals. This work is also helping train the next generation of Native archaeologists, who are increasing in number despite structural barriers in access to education and financial support (Mills and Kawelu 2013). Native American tribes are also leaders when it comes to using archaeology to reclaim heritage so that it can serve tribal needs.

In addition to using THPOs and regulatory contexts to benefit from archaeology, Native Americans are also at the forefront of forcing archaeologists to rethink archaeological methods, practice, and theory (Atalay 2006, 2012; Colwell-Chanthaphonh and Ferguson 2004, 2008; Schneider and Hayes 2020; Watkins 2001). Indigenous archaeology proposes archaeology on Indigenous heritage sites should include Indigenous archaeologists, or Indigenous input, and be interpreted through Native cultural knowledge. This forces a rethinking of the entire archaeological endeavor as archaeology has spread throughout the world. Collaboration with non-white communities provides access to other ways of knowing that can enhance interpretations of the past (Murray 2011). Indigenous archaeology can also lead to research questions that are more relevant to local communities, strengthening connections to archaeological sites.

The Indigenous archaeology movement is not limited to the United States. Collaborations between archaeologists and Indigenous communities have also been conducted in hopes of counteracting the legacy of colonialism and compelling archaeologists to change their practice (Atalay 2006, Murray 2011; Watkins and Ferguson 2005). For example, in New Zealand, archaeologists are opening dialogue with indigenous communities when conducting cultural heritage management (CHM) on Indigenous sites (Allen and Philips 2010). Indigenous input is critical for appropriately understanding sites created by groups with completely different worldviews like the Maori and Moriori (Rika-Heke 2010; Solomon and Forbes 2010). The goal is cultivating a more inclusive archaeology that better serves communities and science. Conversations between Australian archaeologists and aboriginal people have revealed the similar problems Indigenous people have in their campaign for repatriation and sovereignty over cultural heritage sites (Birt and Copley 2005). As is the case of the United States and elsewhere, aboriginal Australians are working to decolonize archaeology and advocating for sites on behalf of future generations (Hemming and Trevorrow 2005; Jackson and Smith 2005). In Brazil, Indigenous peoples are moving to manage cultural heritage sites on their lands, which requires collaborations with contract archaeologists (Silva 2015). These collaborations are also pushing archaeologists to rethink their research questions, re-evaluate the way sites are managed, and come to new understandings of memory (Bezerra 2012; Carvahlo and Funari 2012; Green et al. 2003). Increasingly, archaeologists are building upon these fruitful collaborations to come to new understandings of the past that are improved by cultural knowledge while also forging a more inclusive practice.

Even more importantly, fruitful collaborations provide a chance for redress. Healing past wounds through research can help descendant communities engage with painful pasts and move towards a more healthful present (Schaepe et al. 2017). While federally recognized Native American tribes have unique provisions in historic preservation laws that are not available to African Americans, the way they have practiced heritage conservation, collaborated with archaeologists, and used archaeology in cultural revitalization movements can serve as a template for African American communities.

The ELPAP not only seeks answers to thought-provoking questions about the past but dares to push against disciplinary values of independence over interdependence, fast over slow archaeology, and institutional needs over community needs. Throughout this article we topple these disciplinary values, illuminating an archaeological praxis rooted in interdependence, slow archaeology, and the centering of community needs. Centering our practice in these tenets has proven effective, though not always easy, toward recruiting and training the next generation of archaeologists from underrepresented backgrounds. By using the ELPAP as a case study, this article contends that effective community engagement begins with a radical shift in project design from its skeletal structure. The text that follows provides a multiscale examination of the ELPAP, expanding the discourse on community archaeology to include community building practices among archaeologists, between organizations, and with communities impacted by archaeological work.

## Interdependence Over Independence

At its core the ELPAP is the seedling of the Society of Black Archaeologists (SBA). Launched in 2011, the SBA was established to create a strong network of archaeologists that advocates to ensure the proper treatment of African and African diaspora material culture, promotes more people of African descent to enter the field of archaeology, ensures community collaborations, raises and addresses concerns related to African peoples worldwide, and highlights the past and present achievements and contributions people of African descent have made to the field of archaeology (Flewellan and Dunnavant 2012). ELPAP was designed in alignment with the mission and the vision of the SBA, specifically around the organization’s desire to center community engaged research and increase the number of archaeologists of African descent in the field. For one, the project provides a space for archaeologists of African descent to collaborate with each other on project design, field and lab methods, as well as writing projects. Work at the Estate provided a means for the documentation, excavation, and subsequent conservation of a unique heritage site and artifacts unearthed related to the experiences of enslaved and later free Africans who lived and labored under Danish colonial rule. Furthermore, work at the site provided middle and high school youth, undergraduates, graduate students, as well as junior and mid-career scholars various opportunities to engage in archaeological work and carve out spaces for themselves within a field known for its lack of racial diversity (Zeder 1997).

Disciplinary norms within academia often force scholars into a project paradigm that looks vertical in nature with one Principal Investigator having the final say on the direction and outcome of a project. For example, large early career grants open to junior faculty in the field, like the National Science Foundation’s Faculty Early Career Development Program, require a sole Principal Investigator for proposals. This reinforces a valuation for “the independent scholar” as a demarcation of academic excellence. Authors of this article, during academic presentations, are often asked questions such as “What part of the project is yours?” Questions like this, and others akin to it, are posed as a means to make a Co-PI’s affiliation with the project eligible and legible for institutional merit review requirements. However, what is reified in this merit review practice is demarcating academic excellence based on a view of individualism predicated on how a scholar can demonstrate how they alone are able to build and carry out a project – or put another way, how a scholar is able to show their dominion over a project. This further demonstrates that current academic structures are inept at supporting and recognizing the necessary forms of collaboration and scholarship needed to develop community-based archaeology (Flewellen et al. 2021; Franklin et al. 2020). Projects rooted in community archaeology require a practice of interdependence between archaeologists and communities that aims to democratize power within research design and execution.

Merriam-Webster defines “principal” as “chief,” “executive officer,” and “one who engages another to act as an agent subject to general control and instruction.” These definitions hold within them the connotation of control over others. Rather than have a lone Principal Investigator for the ELPAP, it was decided early on that the core team members Dunnavant, Flewellen, Jones, Odewale, and White would all be co-PIs rotating the title of PI as a practice of democratizing power among themselves. This also served as a means for those who needed the title of “PI,” as it related to their institutional obligations, to assume the title as needed. The foundation of the project, while conceptualized by Dunnavant and Flewellen as part of a larger SWP initiative, was built and fleshed out by the five core team members. In this way, the ELPAP team members jettisoned the idea of independence and worked towards a practice of interdependence where each member acted as a root for the project’s sustainability and growth.

During the second trip that Dunnavant and Flewellen took to the island of St. Croix in October of 2016, they accompanied members of the SWP and DWP to talk with community members about the kinds of heritage work they would like to see on island as well as visit potential maritime and terrestrial archaeology sites for a collaborative project (these community meetings are discussed in detail below). In 2016 two community meetings took place hosted by the SWP that consisted of schoolteachers, directors of nonprofits that catered to the needs of the Afro-Crucian community, and heritage professionals involved in local initiatives as well as staff from the National Park Service. It was during these meetings that SBA, SWP, and DWP affiliates learned more about the ongoing cultural stewardship initiatives that were led by Crucian community members. For example, Crucian Heritage and Nature Tourism organization (CHANT), owned by a tenth generation Afro-Crucian woman, has been training youth on the island in historical woodworking techniques so they may gain employment opportunities in architectural preservation for several years.

Dunnavant and Flewellen visited several potential sites for a terrestrial archaeology project including plantation estates and a former prison. The Estate Little Princess, owned and operated by The Nature Conservancy (TNC), was the last site that the duo visited during that trip. Dunnavant and Flewellen joined representatives from DWP and the SWP on the wrap-around porch of the former eighteenth-century “Great House” at the Estate Little Princess and talked with Kemit-Amon Lewis, who at the time was one of the only Crucian staff members born and raised on the island. Lewis shared details about the environmental work TNC had been doing on the island for more than two decades, yet historical cultural preservation of the grounds, specifically where the enslaved and later free labors of the former sugar and rum distillery lived, was not an area explored in-depth. While The Nature Conservancy has a directive to protect and conserve natural heritage, there are often few if any resources to conserve cultural heritage resources on property under their jurisdiction. This is of particular importance considering TNC is the second largest landowner in the United States. However it should be noted that TNC did fund historian, George Tyson, to conduct archival research on the site for display boards that, prior to the 2017 hurricane season, were open to the public in a corner of the classroom space operating inside the former “Great House.”

The Estate provided many spaces of possibility for an archaeological project that tied together both cultural and environmental preservation needs, which at the time was a priority of the SBA and DWP. Once it was decided that the ELP was a site worth pursuing for an archaeological project, Dunnavant and Flewellen, centering the desires expressed during community meetings hosted by the SWP to train local students in cultural stewardship through archaeology, began to outline a project design. Similarly, the desires of the SWP and the mission and vision of the SBA were politically aligned, focusing on localized capacity building and community interest in highlighting the experiences of people of African descent. The two envisioned a project rooted in education and training and together they enlisted SBA members as co-conspirators. Dunnavant and Flewellen had experience in the nuances of plantation, household, and Caribbean archaeology but realized a project of this scope would require additional talent. The two reached out to Alexandra Jones, Alicia Odewale, William White, and Antoinette Jackson. Alexandra Jones is the founder and CEO of Archaeology in the Community (AITC), a non-profit organization dedicated to K-12 educational programming centered on archaeological theory and method. Collaboration with AITC provided the means for a sustainable youth training component to the project (discussed in-depth below). Alicia Odewale at the time had worked on St. Croix for five years, having completed her dissertation on material remains recovered from the National Park Service site Fort Christiansvaern. Odewale provided regional expertise and, as the project progressed, expertise on lab methods. William White, who has managed and operated dozens of cultural resource management projects on the US mainland since 2005 added significant expertise to field methods at the site. Jackson, known for her work collecting oral histories for the National Park Service, conducted a one-day oral history workshop for students who participated in the youth archaeology field school during the inaugural 2017 field season.

The Co-PIs of the ELPAP aimed to democratize power within the project; no one person controlled any one aspect. Every detail, including what information would be documented on field forms, survey methods for the extensive shovel probe survey of the enslaved village area, as well as whether artifacts should be bagged in paper or plastic bags was discussed as a team. It was thrilling at times, for example experimenting with both paper field forms and White’s Codifi digital field forms. It was also exhausting at times, with nights that left project members flustered as we attempted to consolidate various methodologies into a succinct project. Communication became key and with regular quarterly check-ins between summer field seasons and daily check-ins during the field season, time and space was always made to iron out details. Additionally, the 2019 field season began the ongoing process of self-evaluation that Co-PIs undertook at the conclusion of the field season. This process of self-evaluation ensured that everyone was able to provide feedback regarding the project highlights, strengths, weaknesses, and opportunities.

What was built between the Co-PIs was a support system that combated what Janice Witt Smith and Toni Calasanti (2005) call “institutional” and “social” forms of isolation that harm scholars of color in academia, by limiting avenues for career growth and usurping feelings of social belonging. While scholars of color, as Smith and Calasanti outline, constantly are faced with stress and violence from external pressure to prove their intellect, through the ELPAP Co-PIs built a support system that validated and valued their expertise in the field. Returning to academia's desire to promote individualism as an atom of academic success as discussed above, Barbara Bagilhole and Jacki Goode (2001:162) remind us that “individualism is the myth while male support systems are the reality.” While Bagilhole and Goode are primarily discussing the differences between women and men in academia and do not factor race into their study, Smith and Calasanti provide an intersectional analysis of experiences specifying that the “male support systems” that are the reality of Bagilhole and Goode’s work are white male support systems, leaving men who are scholars of color isolated as well as their female counterparts, although to varying degrees. Rather than subscribing to individualism or the independent scholar as the marker of success, Co-PIs find strength in interdependence that is predicated on a notion of community-building. It is this space of community-building among ourselves that lays at the foundation of how we engage communities impacted by our archaeological work through the ELPAP.

## Community Needs over Institutional Needs

Community archaeology, in practice, is not always easily implemented. Atalay (2010) reminds us that not all aspects of a project will lend themselves to wider participation and not all communities will want to be engaged. Moreover, notions of “community” shift overtime and communities are continually reconstituting themselves (Agbe-Davies 2010; Marshall 2002; Sen 2002). However, “what is most important is that power-sharing with community partners remains central throughout the process” (Atalay 2010:423). This idea of power sharing disrupts disciplinary, colonial notions of authority that have been historically held by archaeologists and democratizes both the dissemination and production of knowledge.

As archaeologists seeking to incorporate the community in all aspects of the project, one must address and recognize all of the communities involved. Identifying the various communities sometimes may be as easy as working with the local town members and leaders; however, in most cases it is very complicated and requires some research into the history of the site. Establishing partnerships or collaborative relationships with the community groups involves dedication and a long-term commitment to the work. Furthermore, for various structural reasons, not all communities are treated with the same historical, social, and political weight. The relationship that develops between archaeologists and marginalized groups is unique. Battle-Baptiste (Battle-Baptiste 2012a, 2012b:101) reminds us that “we must be persistent, patient, and committed to engaging from the beginning with the descendant communities when constructing our research agendas.”

When engaging in community archaeology, critical theorists reflect on their motivations for conducting the project, how their personal experiences influence their knowledge base, and how both are reflected in their interpretations of a site (Leone 1986; Leone et al. 1987; Palus et al. 2006; Potter 1994; Wilkie and Bartoy 2000; Wylie 1985). Critical archaeologists are keenly aware that “all knowledge serves interests” (Potter 1994:36). In understanding that basic principle, archaeologists acknowledge the social ideologies that were governing the past and influence the present. Those ideologies silenced many key players in the past, yet through archaeology those key players are revealed.

Archaeologists have an obligation to the archaeological record as well as the communities the research represents. Archaeology can revise the history of the Americas in a way in which marginalized people and the individuals who built and contributed to the development of modern nations can be celebrated and written into the history. Through conducting community archaeology, the community is no longer the “research subject” but a partner in research. The idea of community as research collaborators comes with many of the same opportunities and challenges associated with adding academic research collaborators to a project including understanding needs, motivations, and unique opportunities that can emerge from such partnerships. However, unlike academic collaborators, there is the added politics of understanding who “speaks on behalf of the community” and how they are compensated for their labor.

Thinking more critically about the role and responsibility of conducting archaeology in the African diaspora specifically, Dunnavant and Flewellen have outlined an *archaeology of redress*. An archaeology of redress speaks to the need for archaeologists of the African diaspora to not only address the needs of the communities in which they work, but also attempt some form of redress for the legacies of slavery that they uncover. The notion that archaeologists have a responsibility to offer some sort of restitution for the historical legacies and traumas they may uncover expands the conceptualization of community archaeology beyond the bounds of the discipline. The idea of redress in archaeology builds directly from the work of Black Studies theorists, community-based participatory research (CBPR), community-engaged archaeology, and public archaeology to posit that the sustainability of the field of archaeology and its relevance in the modern world cannot be disentangled from the sustainability of the communities impacted by the archaeological work. In communities tied to historical injustices - specifically those more vulnerable due to their social location - archaeological praxis requires tending to legacies of inequity; it requires redress. Dunnavant and Flewellen use the archaeological work at the Estate Little Princess and the coral restoration work with Diving With a Purpose as a case study; however, redress extends beyond environmental work into capacity building and other forms of restitution.

## Community Archaeology in Practice in St. Croix

**Fig. 1:**
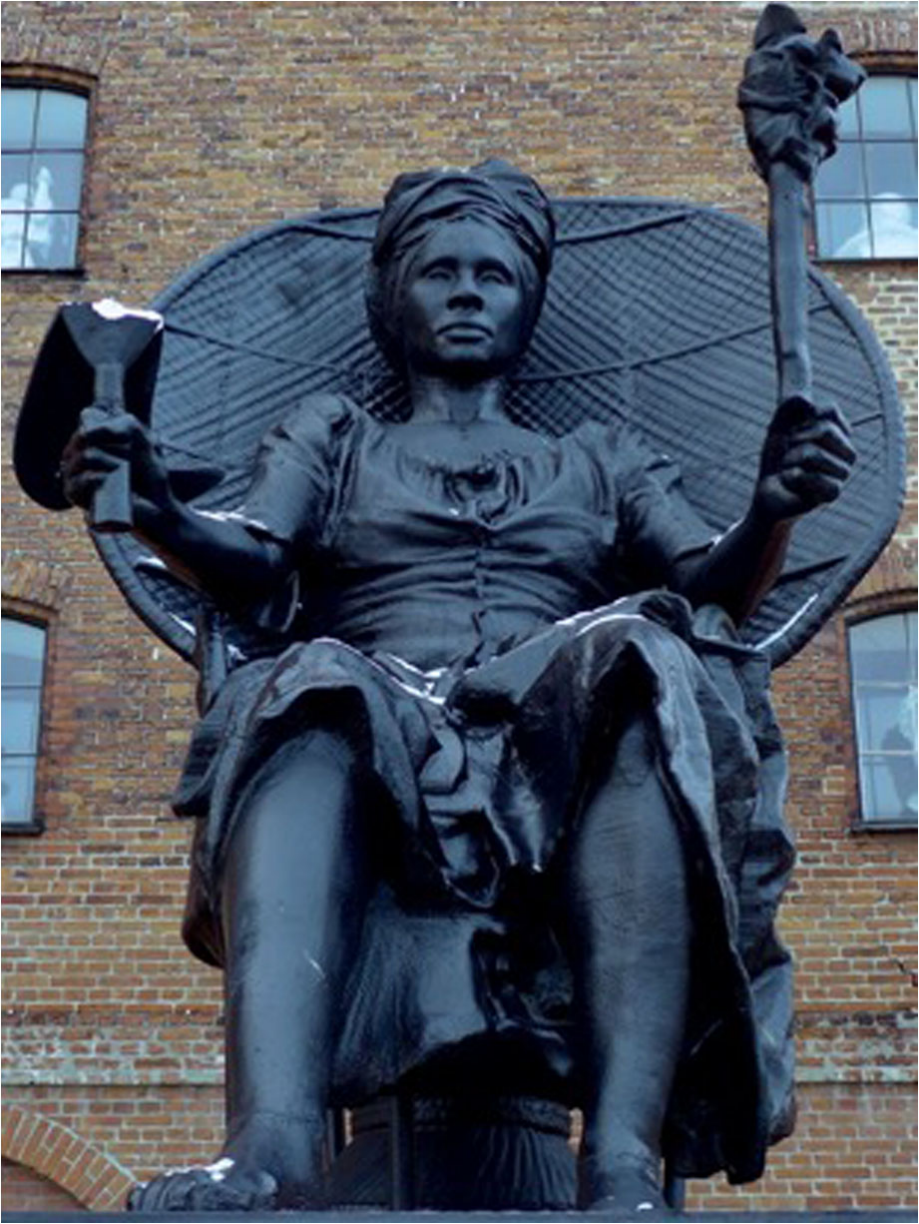
"I am Queen Mary"; La Vaughn Belle and Jeannette Ehlers (2018); photographed in Copenhagen, December 2018. Creative Commons from https://commons.wikimedia.org/wiki/File:I_am_Queen_Mary_(Copenhagen).jpg, accessed July 2020

Archaeological excavations at the Estate Little Princess in 2017 coincidentally coincided with the centennial anniversary of Transfer Day, the day the US Virgin Islands transferred from Danish to US control. The event sparked international conversations around the legacy of Danish colonialism and the role of the United States in current island affairs. The Danish government and Royal Library hosted a number of events, commemorations, and conversations, including a large-scale digitization and on-going translation of historical documents and photographs of the Danish West Indies during the colonial period. The occasion also reinvigorated conversations around other forms of Danish colonialism that persisted in Ghana and India and still persist in Greenland. Works such as the installation of the “I Am Queen Mary” statue in Denmark (Fig. 1), Afro-Virgin Islander artist LaVaughn Belle’s *Cuts and Burns* series, and the documentary “We Carry it Within Us: Fragments of the Shared Colonial Past” by Helle Stenum, a Danish scholar, all brought this history of Danish colonialism to the forefront of public discourse.

**Fig. 2:**
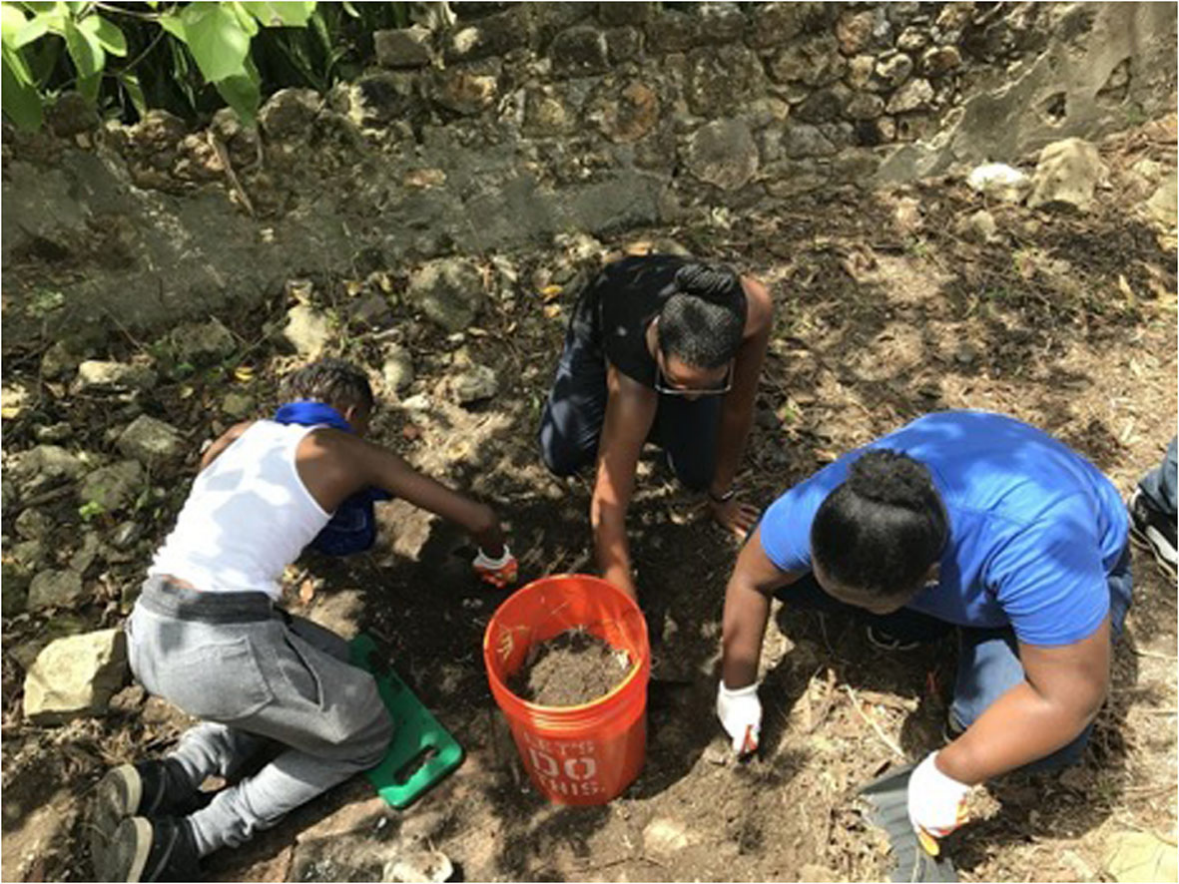
Youth Field School (2018)

Engaging the Crucian community is impossible without engaging its complex history. Numerous books have been published by historians inside and outside of the Virgin Islands, chronicling the stream of colonial powers who have claimed ownership over what’s commonly referred to as the “nation under seven flags” (Dookhan 1994; Hall 1992; Lawaetz 1991; Lewisohn 1970). But fewer scholars have considered the impact that these outside forces have had on the island’s present-day residents, not to mention the pre-Columbian past which is often ignored in modern histories of the island. Jeannette Allis Bastian’s (2003) classic text *Owning Memory: How a Caribbean Community Lost Its Archives and Found Its History*, highlights the ongoing struggle within the Crucian community where limited access to archival records limited the ability to construct, reinforce, and protect one’s own history and collective memory. Bastian (2003:47) discusses how archival collections that are siphoned off and controlled by colonial powers, who position themselves as the “keepers of primary knowledge,” uphold an imperialist agenda. In the case of St. Croix, we know that the island has been occupied by six colonial powers in the past and is currently considered a US territory. However, a majority of the archives, composed of primary accounts and historic records, remain scattered between Denmark and the United States, with a limited number of resources held within local archival collections on the Virgin Islands.

This is the historical context that underpins why present-day community members in St. Croix keep their archives, oral traditions, and all forms of tangible and intangible heritage under close guard with a clear resolve to protect the island’s cultural resources from outside exploitation. Local access to archival records has changed dramatically since Bastian brought this issue to the forefront in 2003; however, both physical and linguistic barriers still exist that impact accessibility to online archival repositories (Flewellen 2019). As the island grows more crowded with tourists, retirees, researchers, transplants, and even archaeologists year after year, it is crucial in our work as Black archaeologists, who are non-locals but committed to community investment, to conduct our work in partnership with local organizations and with a goal of disrupting cycles of exploitation.

Since most archival records and archaeological research preserve events from the perspective of white European males and silence the voices of subjugated persons, those who are committed to telling the stories of the oppressed must work to undo this pattern of erasure (Fuentes 2016). Our focus on community engagement and empowerment is rooted in the need to centralize the needs of both past and present Crucian people, while providing another lens with which to view their experiences outside of the historical white European male gaze. Centering the voices of cultural leaders within the Crucian community, one concern kept rising to the surface regarding people who are not local to the island who have proclaimed themselves to be “experts” in Virgin Island history. The desire by the Crucian community to have their stories told by local individuals is heightened by the fact that members of this community have historically been excluded from having a voice in who controls island territory. In addition to having no control over past ownership of the island, they were also excluded from taking primary ownership over their own archives, historic accounts, and at times the narrative of their own culture. This historic trend of exclusion, erasure, and outside ownership of Crucian history is the root cause that in the past bred a level of distrust between community members and researchers. This is the violence that we as Black archaeologists committed to community empowerment are working to undo, but thankfully there are many other initiatives with the same mission in mind.

The Danish National Archives’ (*Rigsarkivet*) recent mass digitization of Danish colonial documents coupled with the creation of the St. Croix Population Database in 2002 by the Virgin Islands Social History Associates of St. Croix (VISHA), directed by renowned local historian, George Tyson, has increased the level of online access to archival collections. In preparation for the 2017 centennial of the US Purchase of the Virgin Islands, The Danish National Archives established a digital repository containing more than five million documents related to Denmark’s role in the Trans-Atlantic Slave Trade, providing online access to documents previously held in reference collections that could only be viewed in person (Flewellen 2019). The St. Croix Population Database was compiled from census data and archival material scattered between the US, the Virgin Islands, and Denmark. It currently consists of 2.1 million records and biographical entries representing over 110,000 of St. Croix’s residents spanning the years 1734-1917 (Odewale 2016). This database offers the most comprehensive collection of historical material related to St. Croix, including slave trade shipping records, lists of enslaved peoples and free persons of color, property inventories, census records, church records, vaccination records, and a host of other data specific to St. Croix’s people and histories. However, while the database exists on the island, issues around accessibility to the general public persist as access to broadband internet as well as hardware and software necessary to view and download files varies across the island.

In spite of the considerable progress made to increase local access to documentary history, most of these digitization efforts have left out the data generated from archaeological research. Even though barriers of language and legibility still exist in relation to the archival documents (Flewellen 2019), the barriers inhibiting community members from accessing archaeological collections are even greater. Most of the archaeological collections recovered from St. Croix, outside of government sponsored projects by either the National Park Service or the Virgin Islands Department of Planning and Natural Resources, rests in the hands of various universities or private collections without a clear plan for repatriation or a digital footprint to virtually connect these objects back to the island. Our work through the ELPAP aims to bring the archaeological knowledge collected from sites of African heritage in the Virgin Islands into the public eye. Doing archaeology on St. Croix with community empowerment in mind requires a slow approach. The slow approach involves the development of new archaeological methods that acknowledge past trauma and have a commitment to redress where transparency, access, and representation are ingrained into project designs.

## Slow Archaeology over Fast Archaeology

While the nature of archaeological research is in itself a slow process requiring time to secure funding and permits, conduct preliminary research, complete fieldwork, analyze cultural material, and publish results, practitioners of slow archaeology assert that this process is not slow enough. This new movement is urging archaeologists to slow this process down even further, placing an emphasis on knowledge production, long-term activism, and taking time to understand both the unique physical environment and complicated heritage of the sites and people we study (Caraher 2013; Carr and Sturdy Colls 2016). Originating simultaneously within both the Classical Mediterranean world (Caraher 2013, 2016, 2019) and war-torn heritage sites in more recent European history (Carr 2010, 2018; Carr and Sturdy Colls 2016), slow archaeology provides a space where sharing archaeological expertise and protecting cultural knowledge is prioritized over the need to collect excavation data as quickly and efficiently as possible. Through this process, the need to proceed with care is heightened by a desire to support ongoing cultural stewardship and heritage preservation at work within sites of sensitive histories and offer training that helps everyone involved understand the significance of their contributions to the overall project, thus including more voices in the work of interpreting cultural material and rendering our discipline more inclusive and accessible (Caraher 2013; Carr and Sturdy Colls 2016).

During our initial community assessment meetings in St. Croix with various individual community members and community organizations, the desire to have educational programming centered on training locals was expressed. Access to quality education in archaeology requires monetary capital. In archaeology, on-the-ground training through archaeological field schools is implicitly required for those interested in pursuing jobs within the field. However, the cost of participating in these field schools provides a great barrier to entry, particularly for students from low and moderate-income backgrounds. For example, a survey of the field school opportunities offered in collaboration with the Institute for Field Research (ifrglobal.org 2020) reveals an average of $4,322.26 for students to attend, not including the cost of airfare (Table 1). In order to subsidize the cost, students are often dependent upon outside grants and fellowships or are only able to participate in the limited field opportunities that pay their students. Even local field schools offered through the IFR can cost around $3,000 for students. Based on this knowledge ELPAP is committed to offering a youth training program free to Crucian youth each year and since 2017 has provided a free five-week field school for undergraduates attending HBCUs. These two programs, built into the fabric of the ELPAP, provide access to quality education and demonstrate the utility and purpose of community archaeology (Jones, forthcoming).

**Fig. 3:**
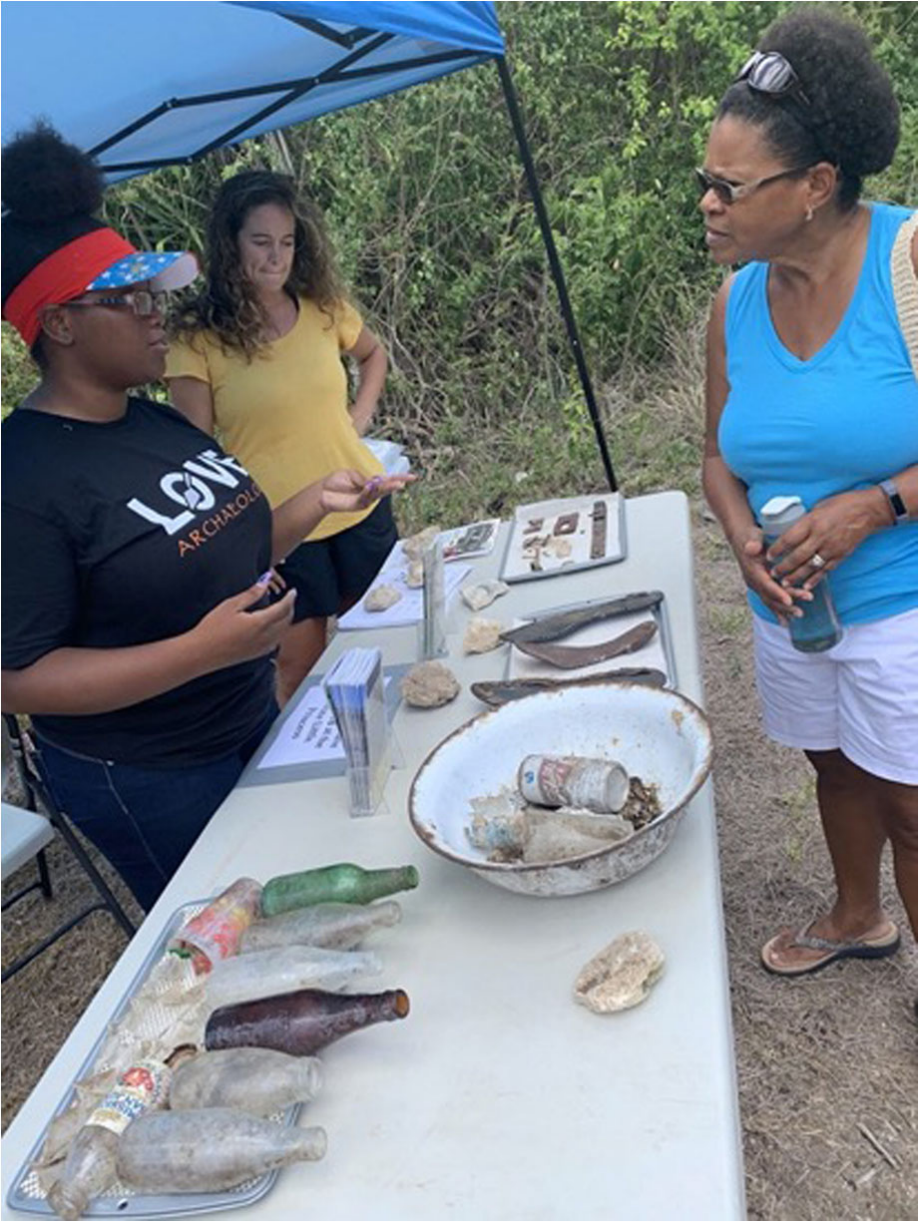
Interns during Public Day (2019)

**Table 1:** Estate Little Princess Artifact Overview, 2017-2020

Name of Field School	Location	Duration (days)	Cost
Colombia: Providence Island	Colombia	27	$ 5,090.00
Bulgaria: Apollonia	Bulgaria	29	$ 4,630.00
Ireland: Ferrycarring (winter and summer sessions available)	Ireland	28	$ 4,500.00
US-OR: Indigenous Archeology	Oregon	41	$ 4,445.00
US-NM: Puebloan Rebels of the Southwest	New Mexico	43	$ 4,500.00
UK: Herefordshire	United Kingdom	34	$ 3,840.00
Bulgaria: Tell Yunatsite	Bulgaria	28	$ 4,210.00
Bulgaria: Varna	Bulgaria	29	$ 4,731.00
Bulgaria: Ancient Mesambria Underwater	Bulgaria	26	$ 5,784.00
US-CO: Amache	Colorado	34	$ 4,150.00
Bulgaria: Pistiros	Bulgaria	29	$ 4,674.00
Portugal: Perdigoes	Portugal	43	$ 4,420.00
Turkey: Bonculklu	Turkey	30	$ 4,365.00
Spain: Cova Gran	Spain	27	$ 4,055.00
Italy: Alcamo	Italy	34	$ 4,710.00
North Macedonia: Excavating Roman Stobi	North Macedonia	28	$ 4,559.00
France: Malvieu	France	27	$ 4,330.00
Italy: Vulci	Italy	34	$ 4,040.00
Israel: Tel Abel Beth Maacah	Israel	13	$ 2,600.00
Ireland: Disert	Ireland	27	$ 4,650.00
Ethiopia: Shire	Ethiopia	27	$ 4,920.00
Croatia: Labor	Croatia	27	$ 4,745.00
Costa Rica: Guanacaste	Costa Rica	34	$ 4,530.00
China: Yangguanzhai	China	34	$ 4,315.00
Denmark: Sorte Muld	Denmark	27	$ 3,870.00
Ecuador: Cochasqui-Mojanda	Equador	34	$ 4,050.00
North Macedonia: Roman Pottery Conservation	North Macedonia	21	$ 3,261.00
Italy: Incoronata	Italy	34	$ 4,445.00
Belgium: Sonian Forest Urban Design	Belgium	27	$ 4,930.00
Pistiros	Bulgaria	28	$ 4,674.00
Underwater	Bulgaria	26	$ 5,784.00
Varna	Bulgaria	28	$ 4,731.00
Cochasqui-Mojanda	Ecuador	34	$ 4,050.00
Moorea	French Polynesia	27	$ 4,300.00
Himalayan Myth and Reality	India	30	$ 4,040.00
Rain Forest Ecology	Indonesia	20	$ 3,820.00
Primate Behavior	Indonesia	19	$ 4,065.00
Birr	Ireland	27	$ 4,825.00
Experimental Archeology Summer	Ireland	27	$ 4,500.00
Ferns	Ireland	27	$ 4,600.00
Bi National Greco Roman Ceramic Conservation	North Macedonia	28	$ 4,673.00
Mosaic Conservation	North Macedonia	20	$ 3,376.00
Pacific Rim	Mexico	27	$ 4,640.00
Sound and City Ethnomusicology	Mexico	27	$ 4,430.00
Nepeña Sessions I&II	Peru	27	$ 4,310.00
Textile Conservation	Peru	27	$ 4,225.00
Ammaia	Portugal	34	$ 4,400.00
Bioarcheology/Nercopolis	Portugal	27	$ 3,960.00
Ruby/Arivaca	US-AZ	34	$ 4,430.00
La Brea Tar Pits	US-CA	25	$ 3,250.00
Mohegan	US-CT	31	$ 3,195.00
Archeological Curation	US-IL	28	$ 4,390.00
Gray Fossil	US-TN	27	$ 2,945.00
Hue Urban Design	Vietnam	27	$ 4,440.00

*In some cases, field schools offered a shorted field session as well. These sessions were removed to not bias the average cost of attendance.

*Accessed on July 2020

## Youth Training Field School

In 2017, the inaugural year, Jones met with the Director of the then Boys and Girls Club, St. Croix, which after the 2017 hurricanes transformed into the Caribbean Centers for Boys and Girls of the Virgin Islands (CCBGVI), to discuss program logistics. The field school was designed for students ages 11-17 that traditionally did not have access to programs of this kind and would be at no cost to the parent. Students were selected from both the Christiansted and Frederiksted branches of the CCBGVI to ensure students from across the island were able to participate in the week-long youth archaeology training program Fig. 2.

The curriculum was designed and implemented by Jones. She developed the curriculum to align with the St. Croix public school system’s high school science curriculum. The students were given two and a half days of lectures which covered the history of slavery in the Virgin Islands, the basics of archaeological excavation, and artifact analysis methods. The students then applied the lessons they learned in the field and the lab during the remaining two days of the program. Kaupp (2001:844) reminds us that “public involvement in archaeological research, including hands-on experience such as excavating and lab work, often leads to support for the value of archaeological investigation and more importantly to a greater understanding of the need to protect vulnerable archaeological sites.” With Kaupp’s words in mind, in addition to teaching the students the theoretical, analytical, and practical sides of archaeology, the students participated in a career workshop and an oral history workshop. Before the students left on the final day, they each participated in a video interview where they were asked questions that forced them to contextualize all of the concepts they learned over the week and then explain why archaeology is important to St. Croix. As archaeologists, if we want future generations to understand and value our work, we have to educate those future generations on the importance of archaeology to them and their community.

The week after the training program in 2017 ended, Jones and Flewellen had lunch with a member of the community. During that lunch the community member asked them an important question: “After the students complete the program, then what?” The students had no opportunities for using the archaeological skills they acquired after completion of the training, as there are very few job opportunities in the field on the island, outside of the limited opportunities for youth provided through the National Park Service, like the Greening Youth Foundation internships and the Youth Conservation Corps. This represented a unique challenge that the team had not initially planned for in the development of the youth program.

The ELPAP project was and is a fluid project constantly evolving based on community needs. One of these was the need for employment based on skills learned during the program. A few changes were made to the youth training to address this concern for the 2018 field season. In an effort to make the program sustainable, with the ability to foster young Crucians’ interest in archaeology and cultural stewardship, a new professional development component was added to the program (internships) and the curriculum was changed to incorporate new archaeological techniques. Two students from the summer 2017 field school were invited back as paid interns for the summer 2018 field season. They worked alongside Jones and assisted in training new student participants. In an effort to expand the knowledge base of the interns, two new lessons were added to the training (nail and ceramic manufacturing techniques). This allowed the interns to not only earn income and build their resume, but also allowed them an opportunity to develop their continued interest in the science of archaeology by learning new skills.

Changes were made to the 2019 youth training based on concerns from the Director of the CCBGVI that the students would be applying for college and/or additional academic programs and would need certificates to verify participation in extracurricular activities. For the 2019 field school, fourteen students from the CCBGVI were selected to participate in the week-long training program and four returning students from summer 2017 and 2018 field schools were invited back as paid interns. Similar to the previous year, new lessons (bottle manufacturing and field conservation) were added to teach the returning interns new archaeological skills. At various times during the field school training, students had the opportunity to talk with some of the ELPAP staff. On the last day of the program the students participated in a career exploration workshop, where the ELPAP staff archaeologists talked about education, career paths, passions, and the meaning of archaeology to them. Understanding representation matters and youth need to see people that look like them in leadership roles in science in order for them to visualize themselves in these fields (Indian University 2019; Rainey et al. 2018). This workshop provided an opportunity for the students to ask questions of the staff members about all aspects of their careers. In addition, they received a certificate of completion and a paid one-year student membership to the St. Croix Archaeological Society, paid for by Archaeology in the Community. As a bonus to the program, students were able to participate in a focus group discussion on the history of the island from their perspective, facilitated by the Slave Wrecks Project. This gave them an opportunity to influence a future exhibition at Fort Christiansvaern curated by the SWP.

The main goals of the program are to train Crucian youth in basic scientific inquiry and to inspire, motivate, and support them in reaching their future goals. Jones reminds us that “Creating scientific literacy among students who have endured multiple intersecting disadvantages may not be glamorous, but it is indeed revolutionary” (Jones forthcoming). The program evolved from a simple field school to a work-training program which seeks to produce young scientists by offering them summer jobs in a career they aspire to pursue one day surrounded by professionals who all teach in colleges and universities to which they plan on applying. Archaeology as a discipline has a history of asserting its desire to contribute to issues of social justice, and at times some have proclaimed that archaeology has the power to change the world (Jones forthcoming; McGuire 2008). Through training youth about the importance of archaeology and how it can be used as a tool for historical revisionism, we are empowering them to explore the history of their shared past.

## Community Archaeology Day at the Estate Little Princess

An important part of the ELPAP is transparency and communication with the larger community. In order to inform the larger island community about the work being conducted at ELP, an inaugural Community Archaeology Day was held in 2019. Flyers were designed and printed then placed in restaurants, coffee shops, and cultural centers all across the island two weeks prior to the event. In addition, emails were sent out to our community partners, local news reporters, and radio stations to disseminate information about the event.

The Community Archaeology Day was held on a Saturday morning from 9:00 A.M. to 12:00 P.M. towards the latter end of the 2019 field season. People visiting the site that day had an opportunity to view excavation units and recovered artifacts that had been excavated during the 2019 field season. Four interns from the youth training were docents for the day. As docents they welcomed visitors, gave them tours of the site, and explained the excavation process and the artifacts associated with the site. This gave the Crucians, community partners, and parents an opportunity to see and learn not only about the site but showcase the knowledge acquired by the students interning with the program Fig. 3.

As mentioned earlier, the success of a sustainable archaeology program varies by project and community. Thus, one of our goals is not necessarily to produce a cadre of archaeologists from St. Croix. Although there is still a persistent need for archaeologists, it is difficult to draw students into a field knowing there are few viable career opportunities that exist on the island. Rather the goal is to produce a cadre of talented young people who have a better understanding and appreciation for both the cultural and natural resources in their communities in whatever field they choose to pursue.

## UC-HBCU

Based on the success of the youth training program during the inaugural 2017 field season, there was a desire and opportunity to offer training and capacity for university students to help extend the pipeline. J. Cameron Monroe along with Dunnavant secured a UC-HBCU grant for the 2018 field season, which provided funding for students from Historically Black Colleges and Universities (HBCUs) to participate in archaeological field schools in St. Croix and Haiti. This grant was later extended for an additional three years with Dunnavant, Flewellen, and White acting as co-Principal Investigators. Due to unforeseen political situations in Haiti in the summer of 2018, the program shifted entirely to St. Croix. As part of the program, students receive a week of intensive training in basic archaeological methods at the Universities of California Santa Cruz and Berkeley and four weeks of excavation, analysis, and mapping experience in St. Croix. The UC-HBCU component is important because it is an attempt to increase the capacity of African Americans in the United States to gain the technical training needed to work in the CRM industry, which is an overwhelmingly white industry tasked with making preservation recommendations for African American heritage sites across the country. An archaeological field school is essential for all professional archaeologists, so providing this opportunity for African American students is foundational to increasing diversity in archaeology.

To date, the program has trained nine HBCU students, with one program alum successfully entering a PhD program in archaeology. Each year, the program has undergone various iterations based on student and faculty feedback. Students are allowed to explore their own research interests alongside faculty and engage in aspects of archaeological fieldwork that are often relegated solely to coordinators. These include liaising with government officials to understand the permitting process, conducting archival research, and assisting with museum exhibition interpretation in collaboration with partners of the Slave Wrecks Project. In both cases of youth and university-level training, the intended goal is not for all participants to become archaeologists but rather to provide them with the experiences needed to become better cultural and natural heritage stewards in whatever careers they pursue.

New opportunities emerge as archaeologists engage communities on their own terms (Agbe-Davies 2010:281). Archaeological work at the Estate Little Princess on the island of St. Croix has affected archaeological practice in other parts of the island as community members are contracting archaeologists for other heritage conservation opportunities. The Crucian Nature Heritage and Tourism organization (CHANT), owned by Frandelle Gerard, an eighth-generation Crucian native, is one such example. Recently CHANT contracted Gabrielle Miller, a Ph.D. student at the University of Tulsa, to conduct excavations at a property they own in Free Gut, a historic neighborhood that housed free Africans during the eighteenth and nineteenth centuries. Miller is working alongside CHANT staff members, co-creating a research design centered around the questions and outcomes CHANT prioritizes. CHANT hopes that the archaeological findings can be used in interpretive signage at the site in the future. Similarly, local students have begun to take advantage of the growing connections between the lead excavators at Estate Little Princess and the Crucian community, as we continue to open new pathways between high school and graduate level training in archaeology. In Spring 2021, we will celebrate Rukiya Andrews, the first Black woman and native Crucian to earn a Master’s degree in archaeology from the University of Tulsa. She assisted in our field school program in 2018 and proceeded to join the Master’s program at Tulsa shortly after to assist in processing collections recovered from her home of St. Croix. Her anticipated return to St. Croix upon graduation is bolstered by her plans to aid in the ongoing recovery effort at local archives with battling post-disaster mold and flooding remediation, as well as her desire to lead interpretation for other sites of Crucian heritage, connecting St. Croix’s rich customs and rituals with archaeological evidence.

## HAHS/DAACS Lab Methods

In accordance with stated guidelines from the VI Department of Planning and Natural Resources, lead excavators along with student participants at both the high school and college levels followed field lab procedures established by Odewale to ensure that any cultural material recovered during the field school and research periods was cleaned and inventoried before leaving the island. In the process of data collection, we discarded any coral or lithic material that had not been culturally modified from all artifact bags. All shell - modified and unmodified - was retained. When present, a sample of architectural material was taken from each unit to avoid overloading the bags with piles of non-diagnostic brick and fragments of modern concrete. It should also be noted that any recovered Afro-Crucian ware remained unwashed to preserve any traces of organic material adhering to the surface of the vessels in support of future testing and residue analysis. To complete the field lab stage, every bag was given the same treatment and first underwent a rough sort, washing, and open air drying overnight before being re-bagged and prepared for shipment to the University of Tulsa Historical Archaeology and Heritage Studies Laboratory.

As previously stated, both the excavation and processing of archaeological material were supported by local and national partnerships to increase local training opportunities but also to ensure transparency in our data management process. The resulting procedure, in which recovered artifacts were processed in public view, on site, alongside students before being temporarily shipped off-island, offers only a temporary solution to the ongoing challenge of the lack of curation facilities or storage space on island. We are continually seeking ways to expand our partnerships and build capacity on island to garner enough financial and logistical support to establish a local curation facility that would eventually standardize the way archaeology is conducted in the Virgin Islands and simultaneously put an end to the consistent stream of archaeological resources leaving the community. However, while we wait for this long-term plan to come into fruition, one way we have been able to make the archaeological material recovered more visible to community members in St. Croix is through another strategic partnership with the Digital Archaeological Archive of Comparative Slavery (DAACS), an organization that shares our interest in standardizing the field of historical archaeology and provides a platform to digitize entire collections for communities to engage with on a much deeper level without any fees attached.

Between the years 2017-19, approximately 14,000 artifacts were recovered from the enslaved laborers’ village at Estate Little Princess. This includes the recovery of 4,280 artifacts during the 2017 field season, 1,668 during 2018, and 7,989 recovered during our most recent 2019 field season (Table 2). Our process to catalog this material has been slow due our desire to develop a new method that would allow us to identify, catalog, and digitize this cultural material at the same time, rendering the collections that are currently off-island more accessible to the St. Croix community. After we launched our partnership with DAACS in 2018, we have spent the last two years focused on receiving DAACS certification in each of the necessary artifact classes, undergoing training in glass cataloging in August 2018 and ceramic cataloging in May 2019. This training will continue in the years to come with more focus on different material types. As we gain more training, our ability to catalog and digitize material simultaneously increases, which also spreads the visibility and potential use of our collections. While we are far from having all of the artifacts from Estate Little Princess entered into the DAACS system, this gradual process with an emphasis on training has led to the development of off-site DAACS training sites and separate DAACS compatible archaeology labs in other states. Another benefit to our work with DAACS is that we are now able to provide training to students beyond our field school program. As the lead researchers for ELPAP branch off into other projects and begin training their own students, DAACS training is helping to standardize the way archaeology is conducted on sites of enslavement and freedom on island. And once these collections from St. Croix are officially live on the DAACS website, this will not only be the first contribution from the Virgin Islands among this consortium of sites but will also be a starting point to new comparative archaeology research avenues between St. Croix and other Caribbean and mainland sites of African heritage.

**Table 2:** Archaeological Field Schools offered through the Institute of Field Research (ifrglobal.org), 2020

Estate Little Princess Artifact Inventory 2017-2019																						
**Years**	**Glass**		**Brick**		**Metal**		**Ceramic**		**Shell**		**Charcoal**		**Fauna**		**Lithic**		**Modern**		**Other**			**TOTAL**
	**N**	***%***	**N**	***%***	**N**	***%***	**N**	***%***	**N**	***%***	**N**	***%***	**N**	***%***	**N**	***%***	**N**	***%***	**N**	***%***	**N**	***%***
**2017**	2280	*53.3%*	78	*1.8%*	1622	*37.9%*	120	*2.8%*	1	*0.0%*	106	*2.5%*	3	*0.1%*	0	*0.0%*	37	*0.9%*	33	*0.8%*	4280	*30.7%*
**2018**	207	*12.4%*	0	*0.0%*	489	*29.3%*	416	*24.9%*	476	*28.5%*	40	*2.4%*	33	*2.0%*	0	*0.0%*	0	*0.0%*	7	*0.4%*	1668	*12.0%*
**2019**	842	*10.5%*	288	*3.6%*	2446	*30.6%*	1190	*14.9%*	2684	*33.6%*	335	*4.2%*	161	*2.0%*	28	*0.4%*	15	*0.2%*	0	*0.0%*	7989	*57.3%*
**TOTAL**	**3329**	***23.9%***	**366**	***2.6%***	**4557**	***32.7%***	**1726**	***12.4%***	**3161**	***22.7%***	**481**	***3.5%***	**197**	***1.4%***	**28**	***0.2%***	**52**	***0.4%***	**40**	***0.3%***	**13,937**	**100%**

In light of the global spread of COVID-19 (coronavirus), we were forced to cancel our field school and excavation plans for 2020. However, due to the virtual nature of our work with DAACS, we continue to make progress even in the face of a pandemic. Over the summer our plan to undergo additional DAACS training to cover metal and other small finds from our excavations was canceled, but the DAACS team has worked hard to continue to provide training opportunities online for our off-site DAACS compatible lab. In May 2020, we received virtual training on clay pipe identification, dating, and cultural interpretation using sample collections from another DAACS site in progress, the Flowerdew Hundred Plantation. The DAACS network of sites available online has become invaluable for continued training and reference material to allow our team to keep moving forward on behalf of our community.

## Conclusion

The Estate Little Princess is a historic property that has not been fully recognized for its value as a heritage site to African diasporic people on St. Croix. In this way, it is an example of how historic preservation and archaeology in the United States overlooks the needs of Black people when it comes to our heritage sites. The ELPAP is an attempt of a group of Black archaeologists to help an African diasporic community reclaim a portion of its heritage. It has become part of a constellation of heritage conservation activities within the Afro-Crucian community that were already underway on St. Croix before the archaeological project was conceived. In this way, the ELPAP makes a contribution to what Chenzira Davis-Kahina (pers. comm.) of the University of the Virgin Islands has called “homegrown heritage and identity conservation.” By connecting with SBA at Estate Little Princess, Afro-Crucian elders are taking sovereignty over some of their heritage sites. In this way, this work is completely organic and local. It comes from a desire to conserve heritage sites, pass cultural knowledge onto future generations, and redress past grievances, omissions, and misinterpretations. The ELPAP and excavations on St. Croix conducted in concert with CHANT are examples of how the local Black community is integrating archaeology into their heritage conservation strategies. Introducing local Black youth to archaeology shows them that archaeology is something black people do, which may motivate some of them to pursue it as a career but also connects them to heritage sites that have been misinterpreted, misunderstood, or co-opted by non-Crucians. The UC-HBCU component not only trains Black students in archaeological method and theory but provides a space for them to connect to African diaspora history in a way that cannot be taught in a classroom. This is capacity building while serving the needs of local non-white communities, a central aspect of indigenous archaeology being applied in a non-Native context. Both indigenous and Afro-Crucians are using the opportunities that come from collaborative, community archaeology projects to expand cultural knowledge and push for heritage conservation activism. The resulting archaeological research is more relevant to local people and the conservation solutions become more attuned to local needs.

Through continued collaboration, ELPAP co-PIs are using the experiences at ELPAP to establish a framework by which others can conduct archaeological research in alignment with the goals and tenets of the Society of Black Archaeologists. Standardized training packets and excavation methods are being developed with the understanding that ELPAP co-PIs and students who go through the program will run archaeological projects at other sites in the future. Toward this end, White has created an SBA excavation manual and collectively, under the directorship of Odewale, we are building a field school manual. An analysis manual is being modified from DAACS’s training protocols. The ELPAP field school was intended to run as a five-year program. As we approach the end of our time there, co-PIs are already beginning to undertake new projects that will mirror the framework executed through the ELPAP, as well as build off of the desires expressed by community members to continue work on the island of St. Croix at different heritage sites. Odewale will continue her comparative research in St. Croix while leading archaeological research within the Historic Greenwood District in Tulsa, Oklahoma; Dunnavant will be investigating the historic community of Africatown just outside of Mobile, Alabama; Jones will continue youth education training at various sites; and Flewellen and White are exploring another plantation site on the island as a comparative study at the request of community members.

Contributing to the success of ELPAP as a team provided us with a number of valuable lessons. Late hours of negotiations over survey methodology forced us to explore our own biases and shed light on the range of methodologies by which we as historical archaeologists have been trained. We interrogated our previous training and collectively established our own best practices that placed community needs over institutional needs and prioritized student training and the democratization of knowledge over individual research agendas. Everything from the shape of shovel test probes to size of screen mesh for sifters was up for consideration. In the end, we understood that methods and field equipment will change as sites and research questions change but we now have a template in place of our own design that we can use for the remainder of our careers.

While the project has proved beneficial to us all as co-PIs, it has also required us to identify and confront the challenges associated with collaboration. In an attempt to understand the true success of the program and identify points of improvements, two post-field school surveys were conducted. One survey directed toward undergraduate field school students assessed their satisfaction with the program and their learning outcomes. The results from that survey revealed that students really benefit from the mentorship associated with having supervisors that shared their cultural and ethnic backgrounds. In terms of improvement, it became clear that students required more structure and hoped to have more time to engage with the interpretation of archaeological material and the site overall. Finally, as with most field school experiences, slight tension arose over the difficulty of clearly defining the position of undergraduate students as collaborators and adults, rather than students that require supervision.

In addition to requesting feedback from student participants, we also took the less common approach of surveying the co-PIs of the project. This component of the project was crucial because it foregrounded a level of reflexivity amongst the co-PIs and allowed us to identify areas for improvement at all levels. The post-field season survey for the coordinators polled co-PIs on everything from the appropriateness of the lodging to the length of the program and gave PIs an opportunity to voice the challenges and strengths of the program anonymously. From the survey we learned that co-PIs overall found the program to be successful at achieving the research and training objectives but there were varying degrees of satisfaction with logistical aspects of the program. Some aspects of the program that need improvement are more clearly defined roles and responsibilities amongst the co-PIs and better communication, including regular debriefs amongst the coordinators.

For those who have run an archaeological field school, it is understood that challenges are a typical part of the process. In preparation for next field season, scheduled for summer 2021 due to COVID-19 cancellations, the co-PIs will review the survey results and determine what steps are needed to alleviate the issues from the previous year. Many academics discouraged us from working with so many collaborators and many academic institutions still disincentivize collaborative research. In addition to the challenges of coordination and the politics of dealing with individual personalities, there is the added struggle of publishing with multiple authors while still ensuring that we as junior faculty retain our own research agendas and solo-author contributions for tenure. However, one of the strengths of this approach - as outlined earlier - is that archaeological work will continue on the island even as co-PIs move on to other projects. Furthermore, the breadth of our expertise has led to various other projects and collaborations outside of ELPAP. Collaborative research is not easy and collaboration with five co-PIs adds another level of complexity, but the ability to galvanize each member's strengths toward training a new generation of diverse archaeologists makes the endeavor more than worthwhile.
